# A comparative analysis of the intestinal metagenomes present in guinea pigs (*Cavia porcellus*) and humans (*Homo sapiens*)

**DOI:** 10.1186/1471-2164-13-514

**Published:** 2012-09-28

**Authors:** Falk Hildebrand, Tine Ebersbach, Henrik Bjørn Nielsen, Xiaoping Li, Si Brask Sonne, Marcelo Bertalan, Peter Dimitrov, Lise Madsen, Junjie Qin, Jun Wang, Jeroen Raes, Karsten Kristiansen, Tine Rask Licht

**Affiliations:** 1 Department of Structural Biology, VIB, Pleinlaan 2, Brussels, 1050, Belgium; 2 Microbiology Unit (MICR), Department of Applied Biological Sciences (DBIT), Vrije Universiteit Brussel, Pleinlaan 2, Brussels, 1050, Belgium; 3 National Food Institute, Technical University of Denmark, Moerkhoj Bygade 19, Soeborg 2860, Denmark; 4 Department of Systems Biology, Technical University of Denmark, Lyngby, 2800, Denmark; 5 BGI-Shenzhen, Shenzhen, 518083, China; 6 Department of Biology, University of Copenhagen, Copenhagen, 2200, Denmark; 7 National Institute of Nutrition and Seafood Research, Bergen, Norway; 8 The Novo Nordisk Foundation Center for Basic Metabolic Research, University of Copenhagen, Copenhagen, Denmark

## Abstract

**Background:**

Guinea pig (*Cavia porcellus*) is an important model for human intestinal research. We have characterized the faecal microbiota of 60 guinea pigs using Illumina shotgun metagenomics, and used this data to compile a gene catalogue of its prevalent microbiota. Subsequently, we compared the guinea pig microbiome to existing human gut metagenome data from the MetaHIT project.

**Results:**

We found that the bacterial richness obtained for human samples was lower than for guinea pig samples. The intestinal microbiotas of both species were dominated by the two phyla *Bacteroidetes* and *Firmicutes*, but at genus level, the majority of identified genera (320 of 376) were differently abundant in the two hosts. For example, the guinea pig contained considerably more of the mucin-degrading *Akkermansia*, as well as of the methanogenic archaea *Methanobrevibacter* than found in humans. Most microbiome functional categories were less abundant in guinea pigs than in humans. Exceptions included functional categories possibly reflecting dehydration/rehydration stress in the guinea pig intestine. Finally, we showed that microbiological databases have serious anthropocentric biases, which impacts model organism research.

**Conclusions:**

The results lay the foundation for future gastrointestinal research applying guinea pigs as models for humans.

## Background

Mammals harbour a large and complex intestinal microbiota, and the impact of the microbial community of the gut on host physiology becomes more and more evident as research in this area progresses. Nevertheless, because of the complexity and the challenges associated with cultivation, understanding of the functions of the mammalian intestinal bacterial community is still very limited (see e.g. [1,2]).

Recently, next generation sequencing techniques have been applied to characterize the intestinal microbiota of humans [3-5] as well as a number of mammalian species [6-10].

Guinea pigs have frequently been used as a model organisms in medical research in the 19th and 20th centuries, resulting in the epithet “guinea pig” for a test subject, but have since been largely replaced by other rodents such as mice and rats. However, there are certain human medical conditions for which guinea pigs constitute better models than other rodents. For example, unlike that of mice and rats, the E-cadherin on the intestinal surface of guinea pigs is homologous to that of humans. This renders these animals one of the most suitable models in studies of infections with the important human pathogen *Listeria monocytogenes*, because E-cadherin serves as the primary receptor interacting with this bacterium upon initiation of intestinal invasion [11], and they are frequently used as such [12-14]. However, only few studies exist, which describe the intestinal microbiota of guinea pigs, and none of these use a metagenomic sequencing approach [15,16]. As recent research indicates that the composition of the intestinal microbiota plays a role in susceptibility to intestinal infections (for a recent review, see [17]), we aimed to elucidate the differences between the microbiota of these commonly used infection models and the humans they are meant to simulate in more detail.

We therefore used Illumina sequencing for characterization of the intestinal microbiota of guinea pigs, and compared our findings to equivalent data from human microbiome sequencing obtained as part of the FP7 MetaHIT project [4]. Here, we present for the first time a gene catalogue containing the prevalent microbial genes in faecal samples from guinea pigs, covering phylogenetic composition as well as main functional categories.

## Results

### Sequencing of the intestinal microbiota of guinea pig

We constructed a non-redundant gene set for all 60 guinea pig individuals comprising 610,834 genes, within which 32.36% (197,687) were complete ORFs (Open Reading Frames) and 67.64% (413,147) were fragmental ORFs. The total data production was 17.57 Gb for eight metagenomic samples (gene libraries), and the average sample size was 2.20 Gb, ranging from 1.97 Gb to 2.94 Gb. A rarefaction curve (Additional file 1: Figure S1a) shows the number of prevalent genes identified with increasing sample number. The extent of sequencing showed that most prevalent *C. porcellus* gut microbiota genes were recovered by our analysis. However, as described later, the taxonomic and functional assignment of these genes proved to be more difficult than for human gut microbiota genes.

In Additional file 1: Figure S1b we compared cross-species assignments to the reference gene databases created for the human and guinea pig metagenome. A relatively large fraction of reads can be assigned to the reference genes of the gene catalogue created for both species, (on average 66.73% of the human samples to the human reference database, and 60.26% of the guinea pig samples to the guinea pig reference database). However, when we switched the databases, the assigned fractions dropped significantly to 9.18% (± 5.34%) of human reads, which could be assigned to the guinea pig catalogue and 6.81% (± 3.45%) of guinea pig reads, which could be assigned to the human gene catalogue.

### Richness and diversity

Rarefaction to 3.5 million reads per sample, where the gene identity of each read was retained, revealed an average gene richness of 353833 ± 29279 for guinea pig faecal microbiome, while the corresponding richness of 402831 ± 89613 genes per faecal sample found for humans was significantly higher (P = 0.037, Additional file 1: Figure S1). However, these estimates were in opposition to the richness observed on most taxonomic levels on PhymmBL taxonomic predictions. Thus, on Species, Genus, and Class level, richness was significantly lower in human samples than in samples derived from guinea pigs (P < 0.05, Additional file 2: Figure S2). Chao1 richness estimates confirmed these results on the above mentioned phylogenetic levels. Functional richness analysis showed the same trends, average COG (Clusters of Orthologous Groups) and KO (KEGG Orthology) richness per sample were lower in the human microbiome, however this was only significant for COG level (P < .001). On all above reported functional and taxonomic levels, Simpson Diversity was significantly higher in human samples than in guinea pigs (P < 0.001), except for phylum level where diversity of guinea pig samples was higher (P = 0.02). Also KO diversity was significantly higher in humans (P = 0.0012).

### Comparison of phylogenetic composition of intestinal bacterial communities in humans and guinea pigs

As described earlier, reads were assigned to reference genomes based on a strict Blast identity cut-off (95%) identity. Using this method we were able to assign 38.2% of the reads obtained from human faecal samples to a given bacterial genome in our database. However, a significantly smaller proportion (9.3%) of guinea pig sample derived reads was assigned to our bacterial reference genome database (Additional file 3: Figure S3).

To directly compare the phylogenetic composition between human and guinea pig gut microbiota, we subsequently used PhymmBL assignments, which allowed assignment of a much higher proportion of reads (75.5% of the Human reads and 62.9% of the Guinea pig reads at phylum level). Data from the eight guinea pig gene libraries (based on 60 animals) were compared with data from human subjects (N = 124) from a recent study [4] sequenced and processed with the same technology as used for the guinea pig samples. Identified abundant bacterial taxons in both hosts are listed in Table 1. 

**Table 1:** Taxons with different abundances in the two hosts

**Taxon**	**Abundance in C. porcellus**	**Abundance in H. sapiens**	**p-value**	**q-value**
Synergistetes	0.14 (0.038)	0.033 (0.018)	2.29E-06	3.08E-05
Chlorobi	1.2 (0.26)	0.31 (0.19)	2.52E-06	3.08E-05
Unclassified	37 (5.9)	25 (3.6)	3.65E-06	3.08E-05
Fusobacteria	0.52 (0.16)	0.14 (0.094)	4.01E-06	3.08E-05
Deferribacteres	0.12 (0.016)	0.048 (0.02)	4.81E-06	3.08E-05
Euryarchaeota	1.8 (0.39)	0.62 (0.36)	7.22E-06	3.77E-05
Cyanobacteria	0.5 (0.088)	0.22 (0.1)	8.25E-06	3.77E-05
Thermotogae	0.21 (0.024)	0.098 (0.042)	1.03E-05	4.12E-05
Chlamydiae	0.25 (0.047)	0.12 (0.061)	2.06E-05	7.18E-05
Chloroflexi	0.06 (0.012)	0.027 (0.012)	2.24E-05	7.18E-05
Chrysiogenetes	0.046 (0.011)	0.021 (0.015)	3.42E-05	9.90E-05
Planctomycetes	0.026 (0.011)	0.0099 (0.0051)	3.71E-05	9.90E-05
Korarchaeota	0.00075 (0.00029)	0.00025 (0.00047)	5.37E-05	0.000132183
Bacteroidetes	15 (3.3)	27 (10)	8.03E-05	0.000183527
Fibrobacteres	0.13 (0.028)	0.058 (0.083)	0.000105854	0.000225823
Spirochaetes	1.2 (0.095)	0.66 (0.35)	0.000123729	0.000247458
Verrucomicrobia	2.1 (2)	0.38 (0.66)	0.000174881	0.000322945
Thermodesulfobacteria	0.0024 (0.00062)	0.0012 (0.0012)	0.000181657	0.000322945
Proteobacteria	9.4 (1.6)	6.4 (2.7)	0.000526935	0.000887469
Nitrospirae	0.032 (0.0076)	0.019 (0.01)	0.000628535	0.001005656
Acidobacteria	0.25 (0.07)	0.16 (0.087)	0.002879726	0.004388154
Deinococcus-Thermus	0.031 (0.011)	0.061 (0.031)	0.004035327	0.005869567
Aquificae	0.016 (0.0037)	0.024 (0.0098)	0.005763463	0.008018731
Firmicutes	29 (6.3)	37 (9.3)	0.015217931	0.020290574
Tenericutes	0.21 (0.093)	0.15 (0.13)	0.020738726	0.025524586
Thaumarchaeota	0.011 (0.0027)	0.0084 (0.0065)	0.020738726	0.025524586
Actinobacteria	1.3 (0.34)	2 (1.1)	0.052286556	0.061969251

Taxons with significantly different abundance (p < 0.05; q < 0.1) or an abundance > 1% in humans and guinea pigs. The average percentage of reads classified to the respective taxon as well as the standard deviation in parentheses is supplied in column 2 and 3.

**Figure 1:**
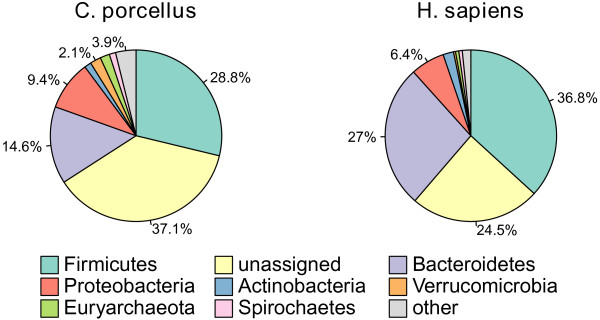
**Distribution of Phyla present in humans and guinea pigs.** Bacterial phyla found in the intestinal microbiota of humans (*H. Sapiens*) and guinea pigs (*C. porcellus*), by PhymmBL annotation excluding unassigned reads. The fraction marked unassigned could not be assigned to a specific phylum.

**Figure 2:**
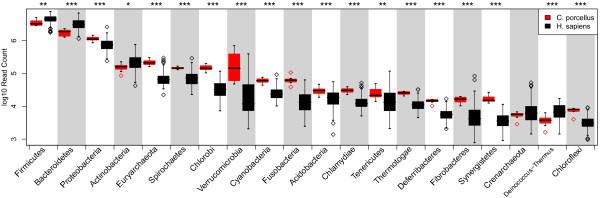
**Most prevalent phyla.** The 20 most prevalent bacterial phyla in guinea pigs (red) and humans (black). Asterisks denote significant differences in abundance, * : q-value < 0.1, ** : q-value < 0.05 and *** : q-value < 0.01. A complete list of the most abundant taxons is given in Table 1.

As in humans, two bacterial phyla, *Bacteroidetes* and *Firmicutes*, dominated the faecal microbiota of guinea pigs (Figure 1). However the distribution of the phyla was quite different in the two hosts, as most of the less abundant phyla were more abundant in guinea pigs than in humans. Only 4 of the 26 significantly different phyla were more abundant in the human gut microbiota than in the guinea pigs (Figure 2). These included the two most abundant phyla, *Bacteroidetes* (q = 0.00018) and *Firmicutes* (q = 0.02). *Actinobacteria* were also more abundant in humans, although this was only suggestive (P = 0.052, q = 0.062). This phylum includes the genus *Bifidobacterium*, that was also most abundant in humans (q = 0.000213) and believed to be important for human health [18]. A complete list of phyla with significantly different abundances in the two hosts is given in Table 1. Thus, in guinea pigs most phyla were more abundant, while in humans the gut microbiota is dominated by fewer phyla. Specifically, the fraction of the total population constituted by *Verrucomicrobia* was five times more abundant in guinea pigs (2.0%) than in humans (0.37%). Furthermore, of the *Verrucomicrobia* found in guinea pigs, 83% were identified as bacteria belonging to the genus *Akkermansia*, while this was true only for 2% of the *Verrucomicrobia* in humans, thus the guinea pig microbiota contains considerably more *Akkermansia* than the microbiota of humans. 

**Figure 3:**
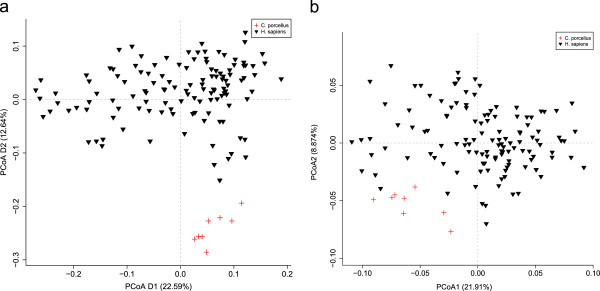
**Bray-Curtis distances between samples.** PCoA (Principle Coordinates Analysis) of Bray-Curtis distances between samples for (**a**) genus and (**b**) KO. A similar separation as shown for the genera was seen on all other taxonomic levels.

The observed differences on genus level are summarized in a PCoA plot (Figure 3`). A similar clear separation between human and guinea pig samples was observed on all phylogenetic levels (data not shown). In total we found 320 of 376 genera to be significantly different between human and guinea pig microbiota (Additional file 4: Table S1). Of these, 225 were more abundant in guinea pig, including genera such as *Methanobrevibacter*, *Desulfovibrio*, while some of the genera known to be important for butyrate formation in the human gut [19] including e.g. *Roseburia* and *Faecalibacterium* were less abundant in guinea pigs. Additionally, many of the genera known to contain human pathogens (*Salmonella*, *Klebsiella*, *Treponema*, *Yersinia*, *Haemophilus*) were also overrepresented in guinea pigs, although low in abundance. This was true also for *Listeria*, mainly due to an overrepresentation of *L. innocua* (data not shown). 

### Comparison of functionality of the human and guinea pig microbiome

Analysis of the functional annotation for the microbiome of both host species revealed that a significantly (p = 0.0014) smaller fraction of guinea pig reads could be assigned to KO’s compared to the human reads. Although statistically significant, the difference between the amount of assigned reads between the two species was very small, i.e. 42.7% of the guinea pig reads were unassigned, while this was the case for 40.5% of the human reads. Overall, the differences between human and guinea pig host were not as extreme as the ones observed for taxonomic composition, however the guinea pig samples still clustered and were defined in their own group (Figure 3b).

Of these KO’s we created a mapping to three different databases. In all of these, a higher fraction of human derived KO’s were assignable to database specific pathways than observed for guinea pig derived KO’s. Thus, we could assign 54.9% of the human reads to SEED pathways [20], while this was true for only 51.9% of the guinea pig KO’s. For the MetaCyc database [21], 14.9% of the human and only 13.6% of the guinea pig KO’s were assignable, and similarly for the KEGG module database [22], where 15.68% of the human and 13.49% of the guinea pig KO’s could be assigned. Due to this bias in assignment, we annotated the reads to eggNOG database, which is based on automatic algorithms to cluster genes of orthologous groups. In this database no significant difference in unassigned reads was detected (27.92% of total reads were unassigned from guinea pigs, while 27.76% were unassigned from humans). It is noteworthy that in the manually created COG database, that is part of eggNOG, the percentage of reads not assignable to COG categories was significantly higher in guinea pigs (43.42%) than in humans (39.46%).

**Figure 4:**
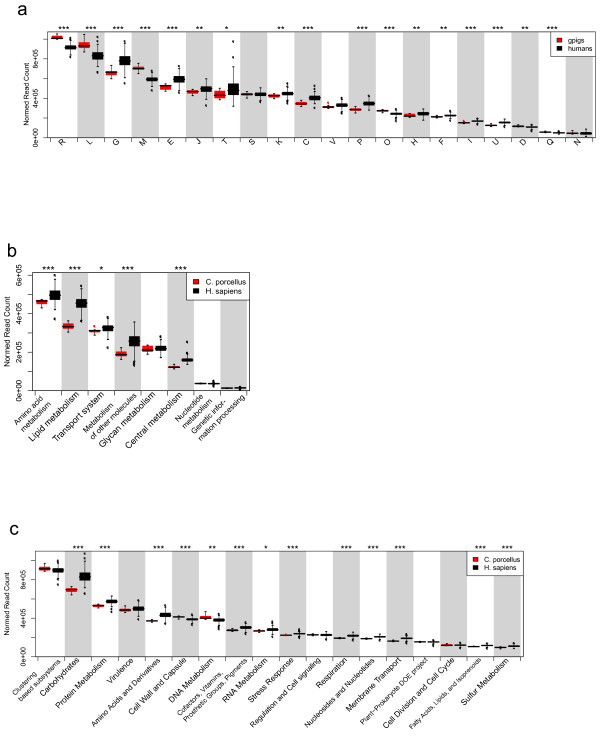
**Functional categories.** Normalized read counts of functional categories from **a**) COG categories **b**) KEGG subcategories and **c**) SEED families, respectively. Asterisks denote significant differences in abundance, * : q-value < 0.1, ** : q-value < 0.05 and *** : q-value < 0.01. COG categories are named as follows: A: RNA processing and modification, B: Chromatin Structure and dynamics, C: Energy production and conversion, D: Cell cycle control and mitosis, E: Amino Acid metabolism and transport, F: Nucleotide metabolism and transport, G: Carbohydrate metabolism and transport, H: Coenzyme metabolism, I: Lipid metabolism, J: Translation, K: Transcription, L: Replication and repair, M: Cell wall/membrane/envelop biogenesis, N: Cell motility, O: Post-translational modification, protein turnover, chaperone functions, P: Inorganic ion transport and metabolism, Q: Secondary Structure, T: Signal Transduction, U: Intracellular trafficking and secretion, Y: Nuclear structure, Z: Cytoskeleton, R: General Functional Prediction only, S: Function Unknown.

Most COG categories were significantly more abundant in human microbiomes, than in guinea pig microbiomes, as predicted by our COG richness estimates. Among the few categories most abundant in guinea pigs were M [Cell wall/membrane/envelop biogenesis], R [General Functional Prediction only], O [Post-translational modification, protein turnover, Chaperone functions], and L [Replication and repair], while most metabolic functions seemed to be more prevalent in the human gut microbiota (Figure 4a, Additional file 5: Table S2).

Similar to this we found that KO’s involved in metabolism were clearly overrepresented in humans on the highest level functional classifications of KEGG modules, but not for KO’s involved in information processing (data not shown). On Subcategory level, lipid and central metabolism were overrepresented in human hosts, and this was also the case for amino acid and most other metabolic pathways (Figure 4b). Noteworthy was that mineral and organic ion transport systems as well as phenylpropanoids, pyrimidine and lipopolysaccharide metabolisms were more abundant in guinea pigs, while monosaccaride transport systems were higher in humans (Additional file 5: Table S2). Among the functional categories covered by SEED, most of the significantly different categories were more abundant in humans than in guinea pig hosts, including mostly metabolic categories. Only few categories were more abundant in guinea pigs, including e.g. “Cell wall and Capsule”, and “DNA metabolism” (Additional file 5: Table S2, Figure 4c). Of the SEED families, 73 out of 112 significantly different ones were more abundant in the human gut microbiota (Additional file 5: TableS2). Most of the human prevalent function were thus related to metabolism, while the functions overrepresented in guinea pigs were more diverse and often related to secondary metabolisms, membrane related and/or transport systems. Of special note is that 6 out of 8 significantly different families belonging to the ‘virulence’ categories were lower abundant in the guinea pig microbiome (Additional file 5: Table S2). In the Metacyc database most pathways were overabundant in human microbiomes (i.e. 27 out of 34 significantly different functions), however, methanogenesis and chemoautotrophic-energy-metabolism involved in hydrogen oxidation, are both overrepresented in guinea pigs. Both these pathways involved in removing H_2_ end-products resulting e.g. from fermentation of polysaccharides, and their overabundance could be a consequence of increased numbers of *Methanobrevibacter* as well as *Desulfovibrio* in guinea pigs (Additional file 4: Table S1).

## Discussion

The data presented here represent the first metagenomic characterization of the faecal microbiota of guinea pigs. The gene catalogue contains all of the prevalent gut microbial genes present in these animals.

It was a logical choice to compare our samples to the human gut microbiota, because this reference data set has been processed with the same sequencing and bioinformatic technologies developed during the European MetaHIT project ( http://www.metahit.eu), which has given rise to several publications on the human metagenome [3,4]. The guinea pig microbiota data presented in this study were thus obtained and processed in a way making them comparable to the human data analysed in the MetaHIT project. However, it should be noted that differences between methodologies for DNA extraction from faecal samples exist between the two studies.

The guinea pig and human gut microbiotas differed substantially on genus and lower taxonomic levels. Human samples as well as guinea pig samples had a fair amount of read matches to reference genes (66.7% and 60.2%, respectively) within the databases specifically created for each environment, however the fraction of assembled reads matching a cross-species database were minor at best (9.2% and 6.8% respectively, Additional file 1: Figure S1 b). This came as a surprise to us, as it shows that the range of strains within the human and guinea pig GI system is fairly restricted and specific to each of these two species. Given that the human samples were obtained from a heterogeneous population spanning two countries (Spain and Denmark), this selectivity of the gut environment across a wide geological range for specific commensal strains is notable. This was further confirmed on species level where we were able to assign 42% of human gut microbiota reads to a reference genome database, but could only assign 10% of guinea pig gut microbiota to genomes within this database. In fact, the human gut microbiota samples did not contain a single sample with as many unassigned reads as any guinea pig sample. While we expected a certain bias in our taxonomic reference database towards gut bacteria within human hosts due to the efforts of the Human Microbiome Project (HMP, http://www.hmpdacc.org/) and the targeted genome sequencing of several typical gut microbes from humans that were included in our reference database, we were surprised by a significant bias also on all functional levels. Not only were we unable to assign the same fraction of assembled guinea pig microbiome reads to KO’s, but these KO’s were more often also not assigned by general purpose functional pathway databases (KEGG, metacyc, SEED). This was also true for eggNOG’s and derived COG categories.

We speculate that the reason for the slightly lower proportion of guinea pig KO’s belonging to defined higher functional categories could be that the guinea pig gut harbors more bacteria that are not well described, than is the case for the human gut. Additionally, a higher amount of environmental bacteria (e.g. from soil) on the feed of the animals may cause a transient presence of soil bacteria encoding functions that are not essential to the gut microbial ecosystem, although this should not bias the general functions databases used. It thus appears that even the gut microbiome of a well-researched mammal living under human observation contains a wealth of undiscovered functionalities.

Herbivorous species (to which guinea pigs belong) typically harbour more phyla and have a higher richness on genus level than omnivores such as humans [10].

Consistently, on most taxonomic and functional levels the observed richness was higher in guinea pigs than in humans, while the diversity of human samples exceeded the diversity of guinea pig samples. Similarly, the lower diversity & higher richness in the KO and COG annotations points to a less specialized gut microbiota with redundant functionality present in many copies throughout the guinea pig microbiota. The guinea pig microbiota is probably more specialized in degrading a more homogenous type of food than the microbiota of the omnivore human host, thus a lower diversity is needed – e.g. the human microbiome may be more diverse because it meets more diverse types of nutrients. When looking at only the two highest level KEGG functional categories, we found that metabolism, but not information processing was overrepresented in the human samples, supporting that the two types of microbiotas differ with respect to the type of materials they metabolize.

Although most higher functional categories were most abundant in human samples, functions related to cell wall and DNA metabolism as well as carbohydrate biosynthesis (Figure 4) are among the few functional categories significantly more abundant in guinea pigs. This might be because the fecal pellets from guinea pigs are much drier than human feces, which will likely require the bacteria to be more robust in sustaining continuous dehydration and rehydration, and thus to e.g. be able to re-synthesize bacterial cell wall components and polysaccharide structures of the outer membrane [23]. Since guinea pigs practice coprophagy (re-ingestion of feces), the gut bacteria will have undergone repetitive cycles of dehydration/rehydration selecting for these traits, as well as for bacteria that can sustain the hostile environment of the stomach and digestive system. However, it should be noted that the SEED family containing traits directly related to desiccation stress was not overrepresented in guinea pigs.

We would have expected an increase in fermentation-related traits in the guinea pig hosts due to the plant polysaccharides in the diet and the increased abundance of H_2_ accepting bacteria (*Methanobrevibacter*, *Desulfovibrio*) in the guinea pig faeces, however in both MetaCyc database and SEED database, “fermentation” was significantly lower in guinea pigs. Given that guinea pigs as herbivorous hindgut fermenters extracts a significant part of their nutritional energy from coecal fermentation of otherwise indigestible carbohydrates, we speculate that this observation may be attributed to a bias within the databases towards omnivore/human fermentation pathways.

While the two most abundant phyla *Firmicutes* and *Bacteriodetes* constituted a relatively large part of the microbiota in humans compared to guinea pigs, the majority of other phyla were more abundant in guinea pigs. Specifically, guinea pig faecal samples contained a much higher fraction of bacteria belonging to the phylum *Verrumicrobia*, most of which (83%) were seen to be represented by *Akkermansia*. This genus is known to contain the species *Akkermansia muciniphilia*[24], which grows preferably on mucin, while only very few other carbohydrates supports its growth [25]. Therefore, *Akkermansia muciniphila* has been suggested to be useful as a biomarker for mucin degradation, and the high amount of *Akkermansia* present in guinea pig faeces might indicate a high turnover of mucins in these animals, perhaps partly resulting from the ingestion of fecal pellets enveloped in mucus gel. If the mucus coating the epithelium is more abundant in guinea pigs, this is relevant in relation to the widespread use of guinea pigs as models for intestinal *Listeria* infections [26,27], since the integrity and thickness of the intestinal mucus layer is known to affect the susceptibility to intestinal infection [28-30]. Additionally, it may be relevant for the design of such studies that *Listeria* were significantly more abundant in guinea pigs than in human samples (P = 1.4*10^-7^) due to an overrepresentation of the non-pathogenic *L. innocua*, probably originating from the commercially available feed given to these animals [13], which was primarily based on plant material likely to be containing *L. innocua*.

This study shows a substantial overlap in phyla inhabiting the human and guinea pig gut. However, on lower phylogenetic levels these environments become less similar. Similarly, the metabolic functions present in the guinea pig samples were different from those in the human samples, suggesting that food breakdown and nutrient extraction is fundamentally different between these two gut ecosystems.

## Conclusions

One conclusion of the presented analysis of the guinea pig metagenome is that on phylum level, it has big similarities to the human metagenome. Therefore, guinea pigs may represent a suitable model for humans in some types of investigations of microbiota-dependent effects including e.g. studies of bacterial gene transfer in the intestinal environment or studies addressing effects of specific feeds or foods on the presence of specific bacterial taxons. However, three major issues have been identified in this study that should be addressed in future studies using guinea pigs as models: Firstly, the huge differences existing between human and guinea pig microbiomes on genus level, as well as the significant differences in the metabolic function of these ecosystems should be taken into account when applying guinea pigs as models for humans in such studies. Secondly, particularly the large amount of *Akkermansia*, probably indicating a large relative amount of mucin in these animals, should be kept in mind when guinea pigs are used as human models in studies where the integrity of the intestinal barrier is of importance, and thirdly, the larger phylum diversity observed in guinea pigs should be taken into consideration when relevant.

## Methods

### Isolation of DNA for sequencing

Faecal samples were obtained during a previous study (Ebersbach et al., 2010), in which a total of 60 guinea pigs were included. 200 mg fresh faeces was dissolved in 1 ml TE-buffer (10 mM Tris–HCl, 1 mM EDTA, pH 8) and centrifuged at 500 x g for 2 min. The supernatant was centrifuged for 5 min at 19,000 x g and pellet was dissolved in 1.2 ml TE-buffer. The sample was transferred to a tube containing 0.5 ml zirconia-silica beads (0.1 mm, Biospec Products) and 30 μl 10% sodium dodecyl sulphate (SDS). Bacterial cells were lysed by shaking for 4 min on a bead-beater (Retsch MM300, VWR International) and centrifuged at 2,300 x g for 1 min. Supernatants were kept at -20°C until further treatment. DNA was extracted using the QIAamp DNA stool Mini Kit (Qiagen) according to the manufacturer’s instructions and stored in 200 μl elution buffer at -20°C until use.

### DNA library construction and sequencing

DNA library preparation followed the manufacturer’s instruction (Illumina). We used the same workflow as described elsewhere [4] to perform cluster generation, template hybridization, isothermal amplification, linearization, blocking and denaturization and hybridization of the sequencing primers. The base-calling pipeline (IlluminaPipeline-0.3) was used to process the raw fluorescent images and call sequences. One whole genome shot gun sequencing library with insert size of 350 bp was generated from DNA of the pooled samples. In total 8 libraries, each containing DNA pooled from 7 or 8 of the total of 60 faecal samples, were sequenced using HiSeq 2000 by 2x75bp pair-end sequencing.

### de novo assembly of Illumina GA short reads

High quality reads were obtained from raw reads by removing adapters, low quality reads and reads that belonged to the host as previously described [4]. Then, the high-quality reads of each DNA sample were assembled by the *SOAP de novo* assembler [31] to contig level. Sequences were processed one by one and the de Bruijn graph data format was used to store the overlap information among the sequences. Overlap paths supported by a single read were unreliable and were removed. Short low-depth tips and bubbles that were caused by sequencing errors or genetic variations between microbial strains were trimmed and merged, respectively. Read paths were used to solve the tiny repeats. Subsequently, we broke the connections at repeat boundaries, and outputted the continuous sequences with unambiguous connections as contigs. The metagenomic special model was chosen, and parameter ‘-K 23’ were used for 75 bp reads. The statistics for this assembly are given in Additional file 6: Table S3.

After *de novo* assembly for each sample independently, we pooled all the unassembled reads together and performed assembly for them in order to maximize the usage of data and assemble the microbial genomes that have low frequency in each read set, but have sufficient sequence depth for assembly by putting the data of all samples together. After assembly, we aligned the reads to the assembled contig and got the ratio of reads assembled. At last, we used all the assembled contigs (including mix assembled contigs) to construct a non-redundant contig set with 90% identity and at least 30 bp overlap using SOAP. Cross database gene alignments were performed with the same parameters, while we randomly subsampled the set of human dataset to 30 samples.

The metagenomic sequences were submitted to NCBI SRA with accession number SRA052638.1.

### Gene prediction and construction of the non-redundant gene set

We use GeneMark to predict ORFs from the contigs assembled from each of the 8 samples as well as the contigs from the merged assembly. The predicted ORFs were aligned to each other using BLAT. Gene pairs with greater than 95% identity and aligned length covering over 90% of the shorter gene were grouped together. The groups sharing genes were then merged, and the longest ORF in each merged group was used to represent the group, and the other members of the group were taken as redundant. Then, the ORFs with length less than 100 bp were filtered out and the remaining ORFs were translated into protein sequences using the NCBI Genetic Codes11.

### Gene taxonomic assignment

Taxonomic assignment of predicted genes was carried out using two different methods: 1) BLASTN was used to assign reads to a reference genome database at a cutoff of 95% sequence identity and > 100 bp overlap. This assignment was used as high confidence assignment on species level. 2) PhymmBL 3.2 with a confidence cutoff of 0.7 [32] was used to assign reads at higher taxonomic levels to get an overview of sample composition on higher taxonomic levels, as PhymmBL employs a probabilistic model to assign a taxonomy to sequences to which no reference can be found in the database and thus allows to map a higher proportion of reads to taxonomic groups than the BLASTN approach that provides very specific mappings, but of a relatively low proportion of reads. As reference database for PhymmBL we used all available reference genomes from NCBI and the set of draft gastrointestinal genomes from the DACC ( http://hmpdacc.org/), both as of the 15.7.2011. The MetaHit reference genes were mapped to taxonomic groups with the same pipeline as described above, care being taken to keep conditions exactly the same.

### Statistical analysis

Each sample (based on microbiota from 7 or 8 animals) was normalized by dividing each feature (species, KO abundance etc.) within a sample by the samples’ respective total sum of reads, and in a second step these percentages were multiplied by the average read count over all samples to retain a sense of sequencing depth in Figure 2 and Figure 4. For the human-guinea pig comparison all samples were treated in the same way. These values between 0 and 1 were multiplied by the average sum of reads over all samples, approximating read number. Feature abundance matrices were transformed by adding 1 to each feature and calculating log10 subsequently, avoiding negative infinite values for absent features. Ordinations of samples were calculated from bray-curtis distances between samples were ordinated by Nonmetric Multidimensional Scaling (NMDS) using the Community Ecology R-package vegan 1.17-9 ( http://CRAN.R-project.org/package=vegan). The data for taxonomic and functional abundances were tested for significant differences using a Wilcoxon rank-sum test that was subsequently corrected for Multiple Testing using the Benjamini-Hochberg false discovery rate (q-value). If not mentioned otherwise, tests were considered significant if they had a p-value ≤ 0.05 and a q-value ≤ 0.1.

Richness was calculated on the rarefied, not normalized, feature abundance matrices on the given level. Rarefaction depth was set to 3.5 million reads per metagenomic sample. From these rarefied matrices we also calculated Simpson diversity [33] and Chao1 richness estimates [34] for the samples. The p-values for richness differences were calculated using a Wilcox rank-sum test on the rarefied richness and diversity measures.

### Gene functional classification

BLASTP was used to search the protein sequences of the predicted genes in the eggNOG 2.0 database [35] and KEGG V55 database [22] with e-value ≤ 1 × 10^-5^ (as in [4]), and the NOG/KEGG OG of the best hit was assigned to each gene. The genes annotated by COG were classified into the 25 COG categories, and genes that were annotated by KEGG were assigned into KEGG modules, MetaCyc modules [21] and Seed pathways [20]. The relative pathway abundance of higher order functional categories were calculated from normalized KO abundance in humans and guinea pigs.

For higher level functional abundance, KO abundances were summed and distributed evenly when KO’s appeared in multiple categories. Functional differences were calculated with a Wilcoxon Ranks-Sum test and multiple testing correction was done by controlling the False Discovery Rate (q < 0.1) using the Benjamini-Hochberg method [36].

## Misc

Falk Hildebrand and Tine Ebersbach contributed equally to the work.

## Competing interests

The authors declare that they have no competing interests.

## Authors’ contributions

FH carried out the bioinformatic analysis, and TE carried out the sampling from guinea pigs. HBN, XL, MB, PD, JQ, JW and JR contributed to the bioinformatic analysis. TRL and KK conceived of the study and participated in its design and coordination together with SBS and LM. TE, FH and TRL drafted the manuscript. All authors read and approved of the final manuscript.

## Supplementary Material

Additional file 1 **Figure S1.a**) Gene rarefaction curve showing that the number of new genes decreases with each sample added. **b**) Comparative assignment of human and guine pig samples to their respective gene catalogue (H-H, G-G) and cross species assignment fractions (G-H, H-G). Click here for file

Additional file 2 **Figure S2.** Sample-wise rarefaction curves for guinea pig and human samples on a) genus and b) COG data. On these two data levels, differences in richness are significantly different on the highest rarefaction depth (3.5*10^6^). Click here for file

Additional file 3 **Figure S3.** Phylogenetic assignment of guinea pig and human metagenomic reads using Blast with an identity cutoff of 95% against bacterial database. Question marks designates reads that were not assignable to a bacterial genome. Click here for file

Additional file 4 **Table S1.** Significantly different genera (q < 0.1, p < 0.05) between human and guinea pig gut microbiome. The depth taxonomic of taxonomic classification is not consistent (due to the PhymmBL algorithm) and this is marked by “?” if the phylogenetic level could not be determined with high confidence. Click here for file

Additional file 5 **Table S2.** Significant functional differences (q < 0.1) between guinea pig and human gut microbiome. These were summarized in five Worksheets: COG categories, KEGG module subsubcategories, SEED categories, SEED families and MetaCyc. Click here for file

Additional file 6 **Table S3.** Assembly statistics for the 8 guinea pig metagenomes, each based on samples from 7 or 8 individual animals. ‘Matched Reads’ designates the number of reads that could be matched to the non-redundant contig set. These were used for subsequent results on functional or phylogenetic characteristics. Click here for file

## Author details

^1^Department of Structural Biology, VIB, Pleinlaan 2, Brussels 1050, Belgium.^2^Microbiology Unit (MICR), Department of Applied Biological Sciences (DBIT), Vrije Universiteit Brussel, Pleinlaan 2, Brussels 1050, Belgium. ^3^National Food Institute, Technical University of Denmark, Moerkhoj Bygade 19, Soeborg 2860, Denmark. ^4^Department of Systems Biology, Technical University of Denmark, Lyngby 2800, Denmark. ^5^BGI-Shenzhen, Shenzhen 518083, China.^6^Department of Biology, University of Copenhagen, Copenhagen 2200, Denmark. ^7^National Institute of Nutrition and Seafood Research, Bergen, Norway. ^8^The Novo Nordisk Foundation Center for Basic Metabolic Research, University of Copenhagen, Copenhagen, Denmark.
