# Supplementary material for: A comparative analysis of the intestinal metagenomes present in guinea pigs (Cavia porcellus) and humans (Homo sapiens)
Source: BMC Genomics. 2012 Sep 28;13:514. doi: 10.1186/1471-2164-13-514 (PMC3472315; doi:10.1186/1471-2164-13-514)
Supplement: Additional file 1 — Figure S1.a) Gene rarefaction curve showing that the number of new genes decreases with each sample added. b) Comparative assignment of human and guine pig samples to their respective gene catalogue (H-H, G-G) and cross species assignment fractions (G-H, H-G). [file 1471-2164-13-514-S1.pdf]

Supplementary Figure 1

a)

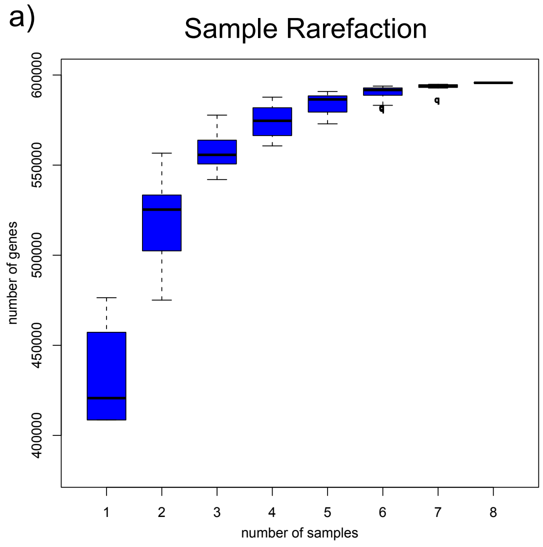

b)

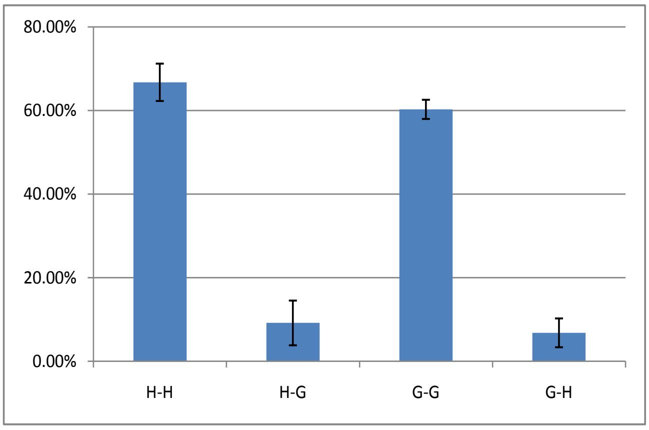

H-H: Human gut microbiome reads map to the 3.3 M human gut microbiome gene set
H-G: Human gut microbiome reads map to the guinea pig gut microbiome gene set
G-G: Guinea pig gut microbiome reads map to the guinea pig microbiome gene set
G-H: Guinea pig gut microbiome reads map to the 3.3M human gut microbiome gene set.
