# Supplementary figures and images for: A comparative analysis of the intestinal metagenomes present in guinea pigs (Cavia porcellus) and humans (Homo sapiens)
Source: BMC Genomics. 2012 Sep 28;13:514. doi: 10.1186/1471-2164-13-514 (PMC3472315; doi:10.1186/1471-2164-13-514)

Supplementary Figure 2

a

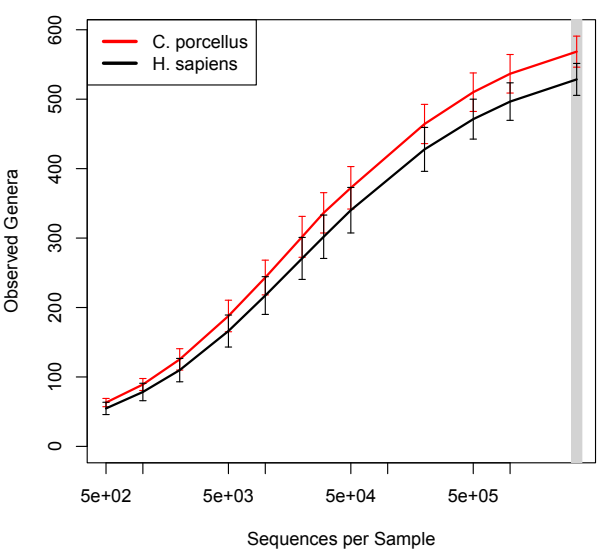

b

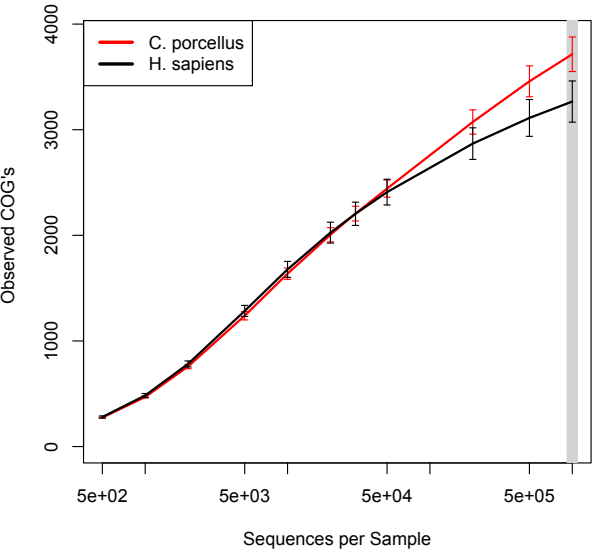

Supplement: Additional file 2 — Figure S2. Sample-wise rarefaction curves for guinea pig and human samples on a) genus and b) COG data. On these two data levels, differences in richness are significantly different on the highest rarefaction depth (3.5*106). [file 1471-2164-13-514-S2.pdf]

Supplementary Figure 3

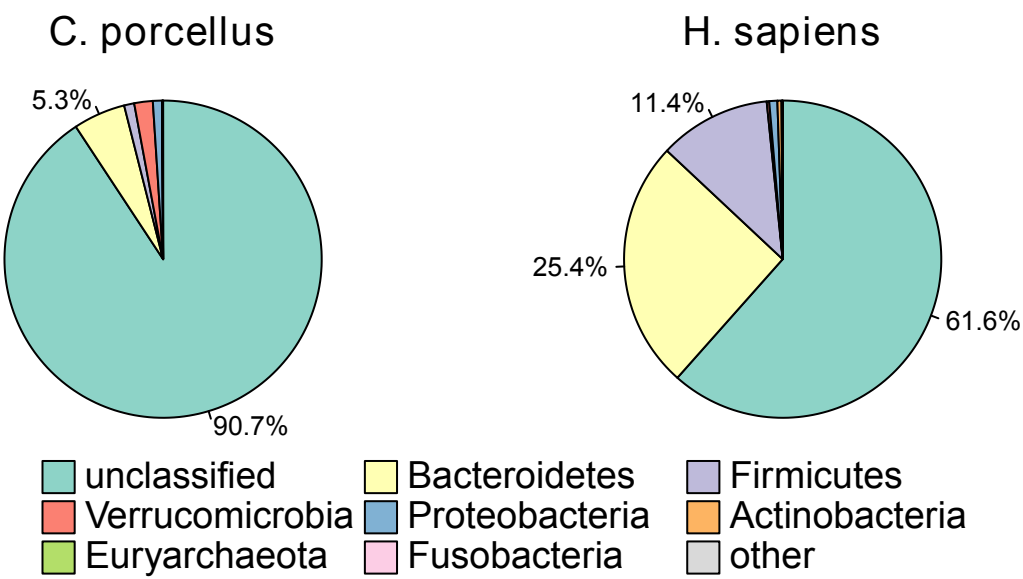

Supplement: Additional file 3 — Figure S3. Phylogenetic assignment of guinea pig and human metagenomic reads using Blast with an identity cutoff of 95% against bacterial database. Question marks designates reads that were not assignable to a bacterial genome. [file 1471-2164-13-514-S3.pdf]
